# FMSP-Nanoparticles Induced Cell Death on Human Breast Adenocarcinoma Cell Line (MCF-7 Cells): Morphometric Analysis

**DOI:** 10.3390/biom8020032

**Published:** 2018-05-23

**Authors:** Firdos Alam Khan, Sultan Akhtar, Sarah Ameen Almofty, Dana Almohazey, Munthar Alomari

**Affiliations:** 1 Department of Stem Cell Biology, Institute for Research and Medical Consultations, Imam Abdulrahman Bin Faisal University, Post Box No. 1982, Dammam 31441, Saudi Arabia; fakhan@iau.edu.sa (F.A.K.); saalmofty@iau.edu.sa (S.A.A.); daaalmohazey@iau.edu.sa (D.A.); maomari@iau.edu.sa (M.A.); 2 Department of Biophysics, Institute for Research and Medical Consultations, Imam Abdulrahman Bin Faisal University, Post Box No. 1982, Dammam 31441, Saudi Arabia; suakhtar@iau.edu.sa (S.A.)

**Keywords:** fluorescent magnetic submicronic polymer nanoparticles, human breast cancer, MCF-7 cell line, anticancer, cytotoxicity, in vitro cell culture

## Abstract

Currently, breast cancer treatment mostly revolves around radiation therapy and surgical interventions, but often these treatments do not provide satisfactory relief to the patients and cause unmanageable side-effects. Nanomaterials show promising results in treating cancer cells and have many advantages such as high biocompatibility, bioavailability and effective therapeutic capabilities. Interestingly, fluorescent magnetic nanoparticles have been used in many biological and diagnostic applications, but there is no report of use of fluorescent magnetic submicronic polymer nanoparticles (FMSP-nanoparticles) in the treatment of human breast cancer cells. In the present study, we tested the effect of FMSP-nanoparticles on human breast cancer cells (MCF-7). We tested different concentrations (1.25, 12.5 and 50 µg/mL) of FMSP-nanoparticles in MCF-7 cells and evaluated the nanoparticles response morphometrically. Our results revealed that FMSP-nanoparticles produced a concentration dependent effect on the cancer cells, a dose of 1.25 µg/mL produced no significant effect on the cancer cell morphology and cell death, whereas dosages of 12.5 and 50 µg/mL resulted in significant nuclear augmentation, disintegration, chromatic condensation followed by dose dependent cell death. Our results demonstrate that FMSP-nanoparticles induce cell death in MCF-7 cells and may be a potential anti-cancer agent for breast cancer treatment.

## 1. Introduction

Breast cancer is the most commonly diagnosed cancer in women and one of the major reasons of cancer death among women. Despite improved precautionary, preventive and treatment strategies, there is no complete cure for the cancer [1,2]. Currently, breast cancer treatment mostly revolves around radiation and surgical interventions; each one of these has its own side-effect issues. Traditionally, the cancer is treated with chemotherapy, hormonal therapy, targeted therapy, and immunotherapy modalities, but all of them carry some side effects and shortcomings [3,4]. The reason for these shortcomings and side-effects are mostly due to non-specificity and systemic toxicity of anti-cancer drugs [5,6,7]. However, the major cause of the failure of current drug therapy, is the high rate of drug resistance of cancer cells [8]. Considering these issues, there is an urgent need for an alternative approach towards cancer management and treatment. Over the past few years, nanoparticles have generated tremendous interest in the area of cancer treatment owing to their precise targeting, biocompatibility, bioavailability, and multifunctional capabilities [9,10,11]. 

Based on their chemical composition, nanoparticles can be broadly classified into two major classes; organic materials which include liposomes, dendrimers, carbon nanotubes, emulsions, and other polymers; and inorganic materials which include metals [12,13,14]. Recently, several attempts have been made to study the effect of different classes of nanoparticles on cancer cells [15,16,17,18,19,20,21,22,23,24,25,26]. (Interestingly, fluorescent magnetic nanoparticles have been used in a wide range of applications in biological systems such as diagnostic, bioimaging, and drug delivery [27,28,29,30,31,32,33,34,35,36,37,38,39,40,41,42,43,44,45] and also in the detection of foodborne pathogens [46]), but there is no report of use of fluorescent magnetic submicronic polymer nanoparticles (FMSP-nanoparticles) in the treatment of human breast cancer cells. In the present study, we tested the effect of FMSP-nanoparticles on human breast cancer MCF-7 cells. We used different concentrations of FMSP-nanoparticles and evaluated their cytotoxic effects by morphometric analysis after 6 h and 24 h post-treatment.

## 2. Materials and Methods

### 2.1. Synthesis and Characterization of Fluorescent Magnetic Submicronic Polymer-Nanoparticles

FMSP-nanoparticles were synthetized and characterized as per method described in a paper published by [47]. Details of the procedure are described below:

Materials: Deionized water (Millipore Milli-Q purification system, Sigma Aldrich, Paris, France) was used in the entire study. Magnetic emulsions and film-forming nanoparticles (Rhodopas Ultrafine PR 3500) were provided by Ademtech SA (Pessac, France) and Rhodia (Aubervilliers, France), respectively. The fluorescent nanoparticles (FluoSpheres F-8787) were purchased from Molecular Probes, (Thermoscientific, Walthman, MA, USA), polyethyleneimine (PEI, Mw = 25,000 g/mole) was purchased from Sigma Aldrich and a modified polyacrylic acid-based amphiphilic graft copolymer (Coatex M883, Mw = 50,000 g/mol, 21 wt%); critical aggregation concentration = 1.4 g/L at pH9 was provided by Coatex, (Lyon, France). The main features of the oil-in water magnetic emulsions are listed in Table 1.

The chemical composition and colloidal characterization were done as per the method described by [48]. The synthesis can be briefly described as follows: an organic ferrofluid composed of iron oxide nanoparticles stabilized in octane by a surrounding oleic acid layer, was emulsified in water with non-ionic surfactants (nonylphenol ether (NP10) and *t*-octylphenoxypolyethoxyethanol (Triton™ X-405) (Sigma Aldrich, Walthman, MA, USA). Table 2 displays the characteristics of the film forming and fluorescent nanoparticles.

#### 2.1.1. Preparation of the cationic magnetic emulsion 

Two milliliters of deionized water were added to either 2 mL (ME1) or 1.2 mL (ME2) of the anionic magnetic emulsion and the mixture was homogenized by vortex mixing. After magnetic separation, the supernatant was removed, and the magnetic droplets were then dispersed in 4 mL of water. Following homogenization and magnetic separation, 2 mL of water was added. After redispersion, this 2 mL of washed magnetic emulsion was added to 4 mL of a PEI solution. After 15 min of stirring, the magnetic droplets were washed two times using 4 mL of water, before final redispersion in 2 mL of water.

#### 2.1.2. Polyethyleneimine adsorption study

The amount of PEI adsorbed onto the magnetic droplets was deduced by titrating the free PEI via specific amine titration using fluorescamine. The fluorescent product obtained was quantified using a fluorescence spectrophotometer (LS 50 system, Perkin Elmer, Boston, MA, USA). A calibration curve was first established (emission intensity measured at 472 nm for an excitation wavelength of 388 nm). The procedure was as follows: PEI standard solutions in milli-Q grade water were prepared, ranging from 0.1 mg mL)_1_ to 1 mg mL)_1_. Standard solution of volume 100 lL was added to 2.9 mL of 0.01 borate buffer pH 9.1, then 1 mL of 0.3 mg mL)_1_ fluorescamine solution in acetone was also added. The PEI amino groups and fluorescamine were then allowed to react for 24 h in the dark. ME1 was used for this study, with all the quantities adjusted to use low volumes (100 lL) rather than 2 mL. The concentration of PEI solutions ranged from C_0_= 0.1 to 25 mg mL)_1_ for a pH between 8 and 11 depending on PEI concentration. After the PEI adsorption step, 100 lL of supernatant was withdrawn and allowed to react with fluorescamine.

#### 2.1.3. Hetero-coagulation step

The film-forming nanoparticles and 10 M NaCl were placed in a 100 mL thermostat reactor. The pH was first adjusted to 7.0 with 1 M HCl solution. The cationic magnetic emulsion (2 mL) obtained in the former stage was mixed with 12 mL of 10 M NaCl (pH 7.0), and 20 mL was continuously added to the reactor content. The temperature was set at 20 °C during the hetero-coagulation process.

#### 2.1.4. Film-formation step

Magnetic hetero-coagulates (magnetic emulsion droplets surrounded by polymer nanoparticles) were separated from the supernatant via magnetic separation and then dispersed in a solution of the amphiphilic copolymer Coatex M883 (0.8 g L)_1_ of 0.01 M borate buffer solution. After a second magnetic separation, the hetero-coagulates were re-dispersed in Coatex M883 solution, then placed in the reactor heated at 50 °C (temperature above the glass transition temperature (T*_g_*) of the film-forming nanoparticles) and stirred for 20 h.

#### 2.1.5. Characterization of FMSP-nanoparticles

The structure and morphology of FMSP-nanoparticles was examined by scanning electron microscopy (SEM) (FEI, INSPECT S50, Brno, Czech Republic), and the size of fluorescent submicron magnetic nanoparticle was measured by transmission electron microscopy (TEM) (FEI, MORGAGNE.268, Brno, Czech Republic). The TEM and SEM analyses were done at the Department of Biophysics, Institute for Research and Medical Consultations, Imam Abdulrahman Bin Faisal University, Dammam, Saudi Arabia.

### 2.2. Treatment of Fluorescent Magnetic Submicronic Polymer-Nanoparticles

MCF-7, a breast cancer cell line isolated in 1970 from a 69-year-old Caucasian woman is a well characterized cancer cell line. The MCF-7 cell line was obtained from Dr Khaldoon M. Alsamman, Clinical Laboratory Science, College of Applied Medical Science, (Imam Abdulrahman Bin Faisal University, Dammam, Saudi Arabia). MCF-7 cells were cultured in a T25 flask in Dulbecco’s modified Eagle’s medium (DMEM) containing L-glutamine, 10% fetal bovine serum (FBS), selenium chloride, 120 U/mL penicillin, and 120 μg/mL streptomycin, at 37 °C in 5% CO_2_ incubator (Heracell 150i, Thermoscientific, Walthman, MA, USA). The cells were then seeded into 96-well cell culture plates to be used for FMSP-nanoparticles treatments. The cells with more than 80% confluence were used for FMSP-nanoparticles treatment and before treatment, FMSP-nanoparticles were autoclaved for 30 min to remove the contaminants. The cancer cells were treated with different concentrations of FMSP-nanoparticles (1.25, 12.5, and 50 µg/mL) and cells were observed after 6 h and 24 h intervals. To obtain an accurate statistical calculation, triplicate samples were considered for both control and treated groups.

### 2.3. Morphometric Analysis

At the end of each treatment (6 and 24 h), the cells treated with different concentrations of FMSP-nanoparticles were removed from the CO_2_ incubator and were observed under an inverted microscope (TS100F Eclipse, Nikon, Tokyo, Japan) equipped with a digital camera. We used 10×, 20× and 40× magnifications to examine the anatomy and structure of both treated and control cells.

### 2.4. 4’,6-Diamidino-2-phenylindole Staining

The 4’,6-diamidino-2-phenylindole (DAPI) staining assay was carried out to examine the effect of nanoparticles on the cell nucleus. Both control and treated cells were pre-treated with the freshly prepared ice-cold (4%) paraformaldehyde. The cells were treated with 0.1% Triton™ X-100 in phosphate-buffered saline (PBS) for 5 min for permeabilization of cell membranes. Then, cells were stained with DAPI with a concentration of 1 μg/mL prepared in PBS for 5 min in the dark environment. Thereafter, the cells were washed with 0.1% Triton™ X-100 prepared in PBS. The cell morphology was analyzed by laser confocal microscope (Zeiss, Berlin, Germany) equipped with a digital camera. The difference between live cells and dead cells were calculated and compared in both control and FMSP-nanoparticles treated groups.

### 2.5. Statistical Analysis

All data were presented as mean ± standard deviation (SD) from triplicate experiments. Statistical analysis was performed using ANOVA followed by Dunnett’s post hoc test of GraphPad Prism software. * *p* < 0.05, and ** *p* < 0.01 were considered statistically significant.

## 3. Results

### 3.1. Characterization of Fluorescent Magnetic Submicronic Polymer-Nanoparticles

**Figure 1:**
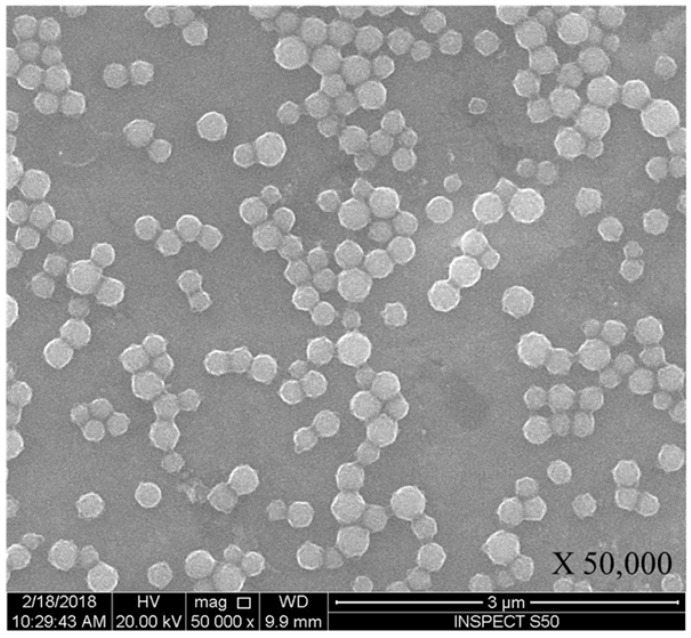
Spherical structure of fluorescent magnetic submicronic polymer (FMSP)-nanoparticles showing through scanning electron microscopy (SEM) with 50,000× magnification.

**Figure 2:**
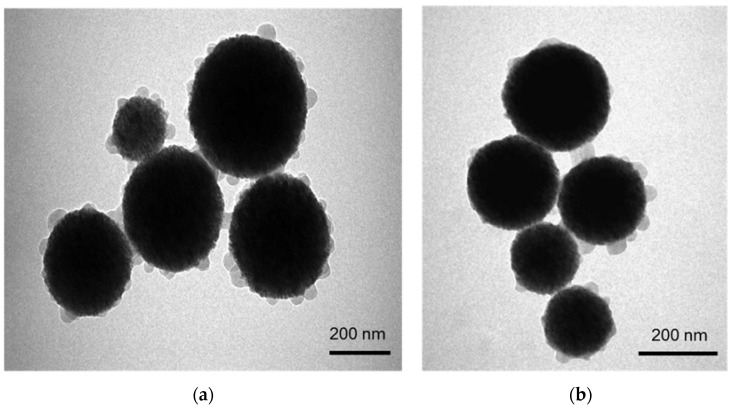
(**a**) shows the structure of FMSP-nanoparticles through transmission electron microscopy (TEM) showing spherical shaped nanoparticles and (**b**) shows the nanoparticles with size ranging from 150 nm to 400 nm.

The morphology, structure and size of FMSP-nanoparticles was determined by using SEM and TEM investigations. SEM analysis showed that nanoparticles were crystallized and spherical in shape (Figure 1); whereas TEM analysis revealed nanoparticles have an average diameter of 100 to 400 nm (Figure 2).

### 3.2. Morphology of the Fluorescent Magnetic Submicronic Polymer-Nanoparticles Treated MCF-7 Cells

**Figure 3:**
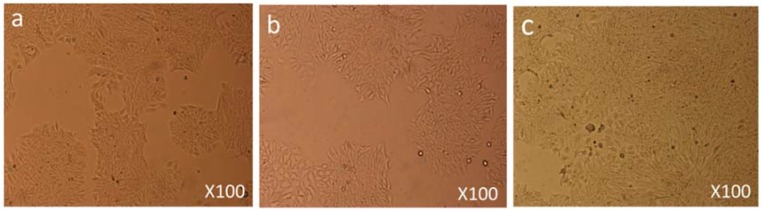
Cell Morphology: The MCF-7 cells showing morphology (**a**) control (non-treated), (**b**) treated with FMSP-nanoparticles (1.25 μg/mL) for 6 h, (**c**) treated with FMSP-nanoparticles (1.25 μg/mL) for 24 h. FMSP-nanoparticles-treated cells did not show any morphological changes when compared to control group cells. 100× magnification.

**Figure 4:**
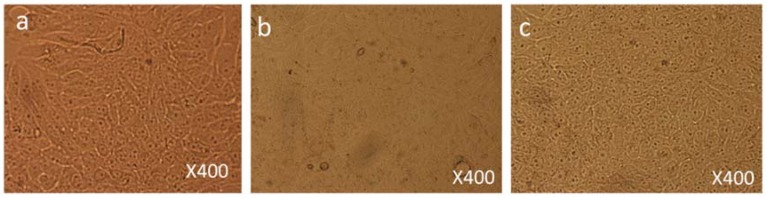
Cell Morphology: The MCF-7 cells showing morphology (**a**) control (non-treated), (**b**) treated with FMSP-nanoparticles (1.25 μg/mL) for 6 h, (**c**) treated with FMSP-nanoparticles (1.25 μg/mL) for 24 h. FMSP-nanoparticles-treated cells did not show any morphological changes when compared to control group cells. 400× magnification.

Both control and FMSP-nanoparticles-treated cells were observed under 100×, 200× and 400× magnifications to study detailed morphological changes. The dose of 1.25 µg/mL produced no cell death when observed under 100× magnification (Figure 3a–c), when observed under 400× magnification we also did not see any morphological changes as compared to control cells (Figure 4a–c). We did not find any difference in cell morphology and structure at both 6 h and 24 h post-treatment.

**Figure 5:**
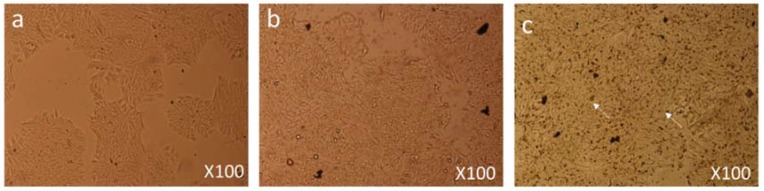
Cell Morphology: The MCF-7 cells showing morphology (**a**) control (non-treated), (**b**) treated with FMSP-nanoparticles (12.5 μg/mL) for 6 h, (**c**) treated with FMSP-nanoparticles (12.5 μg/mL) for 24 h. FMSP-nanoparticles-treated cells showing cell death (arrows) after 24 h of post-FMSP-nanoparticle treatment. 100× magnification.

**Figure 6:**
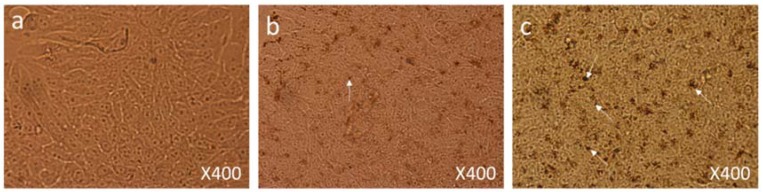
Cell Morphology: The MCF-7 cells showing morphology (**a**) control (nontreated), (**b**) treated with FMSP-nanoparticles (12.5 μg/mL) for 6 h showing beginning of cell death (arrows), (**c**) treated with FMSP-nanoparticles (12.5 μg/mL) for 24 h. FMSP-nanoparticles-treated cells showing high level of cell death (arrows) after 24 h of post-FMSP-nanoparticle treatment. 400× magnification.

When MCF-7 cells were treated with a dose of 12.5 µg/mL, moderate morphological changes in cell morphology and structure were observed 6 h post-treatment under 100× magnification (Figure 5b) as compared to the control group cells (Figure 5a). When cells were observed 24 h post-treatment, cell death had occurred in a major part of the culture plate (Figure 5c and Figure 6c).

Under 400× magnification, dead cells and their debris and cells with nuclear augmentation were observed (Figure 6c).

**Figure 7:**
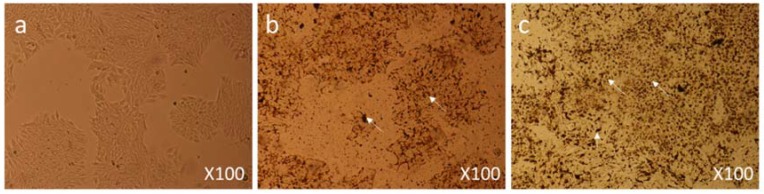
Cell Morphology: The MCF-7 cells showing morphology (**a**) Control (nontreated), (**b**) treated with FMSP-nanoparticles (12.5 μg/mL) for 6 h showing high level of cell death (arrows), (**c**) treated with FMSP-nanoparticles (12.5 μg/mL) for 24 h showing drastic increase in the cell death (arrows) after 24 h of post-FMSP-nanoparticle treatment. 100× magnification.

MCF-7 cells were treated with a dose of 50 µg/mL, 6 h post-treatment they showed significant morphological changes in cell structure and numbers (Figure 7b) as compared to control group cells (Figure 7a). After 24 h post-treatment, cells were observed under 400× magnification; extensive damage in the cellular structure and contents of the nanoparticles-treated cells could be seen (Figure 7c).

**Figure 8:**
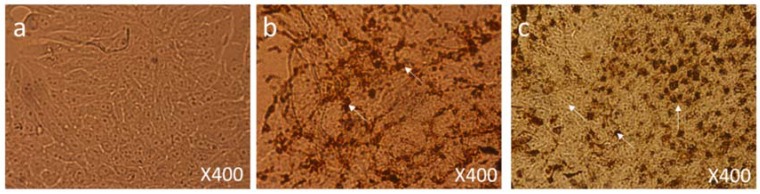
Cell Morphology: The MCF-7 cells showing morphology (**a**) control (nontreated), (**b**) treated with FMSP-nanoparticles (12.5 μg/mL) for 6 h showing high level of cell death, nuclear disintegration, nuclear augmentation (arrows), (**c**) treated with FMSP-nanoparticles (12.5 μg/mL) for 24 h showing drastic increase in cell death, nuclear disintegration, nuclear augmentation (arrows) after 24 h of post-FMSP-nanoparticle treatment. 400× magnification.

We also found many dead cells and their debris (Figure 8c) compared to the control group cells (Figure 8a). In addition, nanoparticle-treated cells showed significant nuclear condensation and nuclear fragmentation (Figure 8b,c).

### 3.3. Cell Death Analysis by 4’,6-diamidino-2-phenylindole Staining

**Figure 9:**
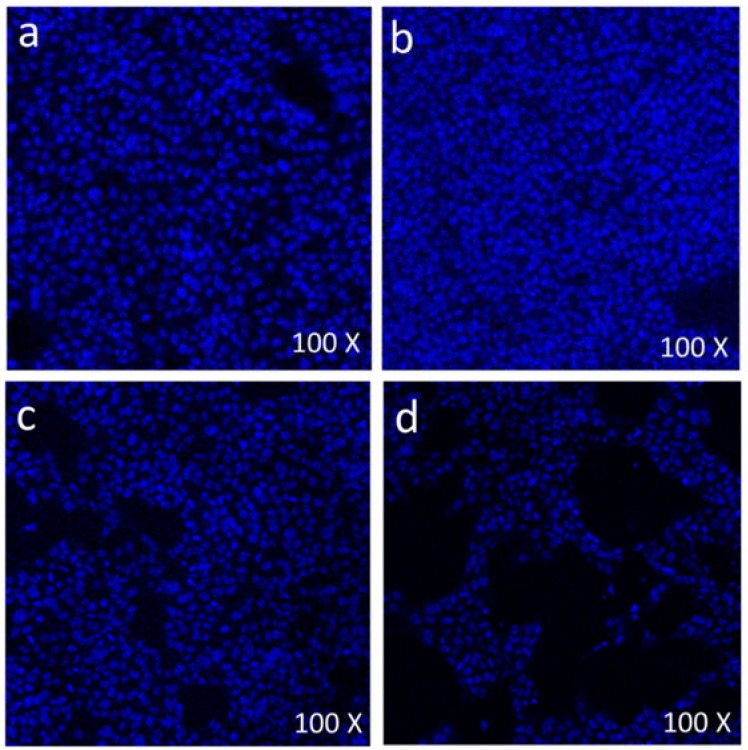
Scanning confocal microscopic images of MCF-7 cells stained with 4’,6-diamidino-2-phenylindole (DAPI) (blue color): The control cells (without FMSP-nanoparticles treatment) (**a**), and with FMSP-nanoparticles (1.25 μg/mL) (**b**), 12.5 μg/mL (**c**), and 50 μg/mL (**d**).

During morphological analysis, a high incidence of cell death was observed, especially with 12.5 µg/mL and 50 µg/mL treated cells. With a view to confirming that the cell death was due to the apoptosis pathway, we stained cells with DAPI. DAPI staining was done to evaluate the effect of FMSP-nanoparticles on the cell nucleus of both control and treated cells. DAPI staining was carried out with dosages of 1.25, 12.5 and 50 µg/mL of FMSP-nanoparticles for 24 h post-treatment. Treatment with a 1.25 µg/mL dose showed no nuclear disintegration (Figure 9b) as compared to control cells (Figure 9a), whereas treatment with 12.5 µg/mL showed a significant amount of nuclear death (Figure 9a). The 50 µg/mL dose showed a highly significant amount of nuclear disintegration and cell death (Figure 9d).

**Figure 10:**
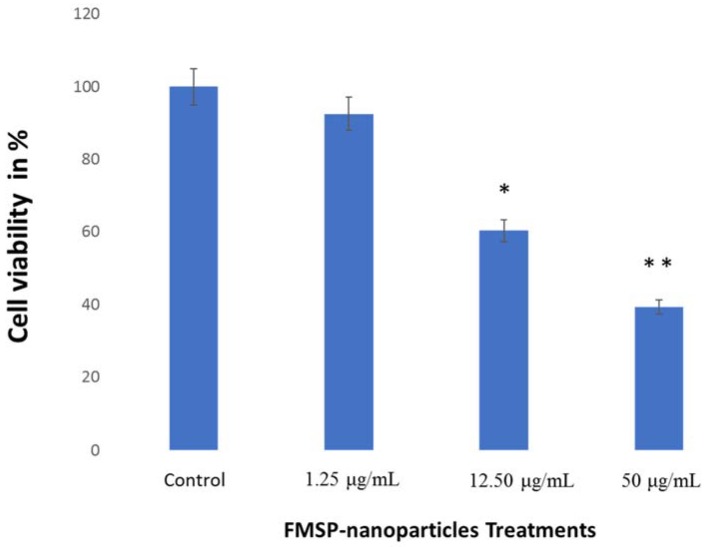
Cell viability analysis. The MCF-7 cells were treated with FMSP-nanoparticles with various concentrations (1.25 μg/mL, 12.50 μg/mL, and 50 μg/mL) for 24 h. Data are the means ± standard deviation (SD) of three different experiments. Differences between two treatment groups were analyzed where *p < 0.05 and **p < 0.01.

We calculated the number of live cells after 24 h of FMSP-treatment. When cells were treated with dose 1.25 μg/mL, it was found that the cancer cells’ viability was 92.50% (Figure 10) but when the cells were treated with 12.5 μL/mL and 50 μg/mL, the cancer cells showed dose-dependent decreases in the cell viability of 39.25% and 60.35%, respectively (Figure 10).

## 4. Discussion

This is the first study in which the morphological and structural changes caused due to nanoparticle treatments has been studied in detail. The morphological analysis with various magnifications (100×, 200× and 400×) of the cells revealed that FMSP-nanoparticles produced dose-dependent effects on the MCF-7 cells. For example, the dose 1.25 µg/mL produced no effect on the cell morphology, whereas dosages of 12.5 µg/mL initiated the cell nucleus augmentation, nucleus fragmentation and cell death. Strikingly, the dose with 50 µg/mL showed a strong effect on both cell morphology and cell death. For example, post 6 h treatment, there was a strong indication of cell death observed as compared to control group cells and clear signs of early nuclear fragmentation. Post treatment at 24 h and 48 h a high degree of chromatin condensation, nuclear condensation and nuclear fragmentation, with the strong presence of cellular debris was observed. The present study is the first of its kind where FMSP-nanoparticles treated cancer cells were morphologically analyzed in detail. Recently, it has been reported that nanoparticle treatment caused cell morphology [49], but most of the studies are confined to the quantitative aspects of nanoparticles. Our findings demonstrate that it is equally important to do a morphological analysis of the cells treated with nanoparticles. The morphological analysis provides information about the extent of nanoparticles effects on cancer cells and their internal organizations. Under high magnification, we could see the extent of the impact of nanoparticles on cancer cell numbers, nucleus and cell membrane. As most of the cancer cells were found to be dead with a dose of 50 µg/mL, it would be interesting to examine the effects of nanoparticles on other cell organelles such as mitochondria, endoplasmic reticulum, Golgi apparatus, and other components of the cells.

Magnetic nanoparticles have excellent characteristics for better target drug delivery [50,51,52,53,54,55] and are an effective way of treating cancer [52]. During the morphological analysis, we found that high doses of FMSP-nanoparticles caused cytotoxic effects on MCF-7 cells, which leads to nuclear disintegration and fragmentation followed by death. Moreover, a high dosage of FMSP-nanoparticles seems to induce MCF-7 cell death via apoptosis that was demonstrated by the results from the DAPI (6-diamidino-2-phenylindole) nuclear staining technique. This staining technique is known to form fluorescent complexes with double strand DNA. The apoptotic nuclei which undergo nuclear fragmentation and chromatin condensation can be stained by DAPI [56,57]. We found that the cells treated with a 1.25 µg/mL dose showed no nuclear disintegration as compared to control group cells. The 12.5 µg/mL and 50 µg/mL dosages caused a significant nuclear fragmentation, and disintegration. Nuclear fragmentation and disintegration are an indication of apoptosis. Similar results have been reported where nanoparticles caused nuclear fragmentation, disintegration and cell death in cancer cells [58,59].

During our study, we also measured the average size of the FMSP-nanoparticles in order to understand how these nanoparticles produced a cytotoxic effect on cancer cells. The SEM and TEM analysis revealed that FMSP-nanoparticles between 100 to 200 mm size caused significant death of cancer cells. While we do not know the mechanism of interaction of FMSP-nanoparticles with cancer cells, the possibility of cell internalization cannot be ruled out. There are several reports of internalization of nanoparticles studied in various cancerous cells [60,61,62] which led to cell death. 

## 5. Conclusions

The present findings confirmed that FMSP-nanoparticles induced cell death in a dose dependent manner in cancer cells. DAPI staining confirmed the cell death due to nuclear disintegration, hence the role of apoptosis induced cell death cannot be ruled out. The dose of 1.25 µg/mL produced no significant effect on the cell death, whereas a dose of 12.5 µg/mL produced significant cell death 24 h post-treatment. Interestingly, a dose of 50 µg/mL caused highly significant cell death during the same period. We have demonstrated that FMSP-nanoparticles are potential biomaterials to be used in the treatment of cancer.

## Figures and Tables

**Table 1:** Features of the magnetic emulsions used.

Reference	ME1	ME2
*D*_h_ (nm) ^a^	246	249
*D*_n_ (nm) ^b^	219	221
D_w_ (nm) ^b^	233	232
Polydispersity index ^b^	1.066	1.050
Solid content (%)	6.1	9.7
Density (g cm^−3^)	2.66	2.42
Magnetic content (wt %)	70	70

^a^ Obtained from dynamic light scattering (DLS); ^b^ Obtained from Transmission Electron Microscopy (TEM) analysis.

**Table 2:** Features of film-forming and fluorescent nanoparticles used.

Reference	Rhodopas PR 3500	Yellow-Green Fluospheres
*D*_h_ (nm) ^a^	47	24
Polymer nature	Acrylic copolymer (*T_g_* 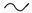 10 °C) ^b^	Polystyren (*T_g_* 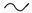 10 °C) ^c^
Density (g cm^−3^)	1.03 ^d^	1.04 ^c^
Fluorescence	/	λ _exc_ = 505 nm ^c^ _em_ = 515 nm ^c^

^a^ Obtained from dynamic light scattering (DLS); ^b^ Obtained from differential scanning calorimetry; ^c^ Reference; ^d^ As per Rhodia SARL (La Défense, France).

## Availability of Data and Material

The data analyzed are available from the corresponding author upon request.
